# A new morphological phylogeny of Malacostraca comparing the application of character dependencies and implied weighting

**DOI:** 10.1111/cla.12611

**Published:** 2025-04-04

**Authors:** Markus Grams, Ambrosio Torres, Christian S. Wirkner, Stefan Richter

**Affiliations:** ^1^ Allgemeine & Spezielle Zoologie Universität Rostock Universitätsplatz 2 Rostock 18055 Germany; ^2^ Centre for Integrative Biodiversity Discovery Museum für Naturkunde – Leibniz‐Institut für Evolutions‐ und Biodiversitätsforschung Invalidenstr. 43 Berlin 10115 Germany

## Abstract

Using a new character matrix composed of revised matrices of previous analyses and new morphological findings, the phylogeny of Malacostraca (Pancrustacea) is analysed anew with 207 characters for 35 terminal taxa across all recognized orders. Particular emphasis was placed on methodological versatility, including different degrees of implied weighting and one of the first applications of methods recently developed in TNT (with the xlinks‐command) for considering character dependencies. With >67% of ontological dependencies our character matrix offers a perfect opportunity for putting this new methodology to the test. In particular, we can demonstrate the significant impact of character dependencies and conclusively argue the usefulness of “xlinks” (or the consideration of character dependencies in general). Furthermore, the multimethod framework also enables a comparative evaluation of established and new approaches, and the resulting cladograms thereof. Although our various results leave many questions about the phylogeny of Malacostraca unanswered, clear support is emerging for some monophyla, whereas some surprising findings give reason for methodological reflection. Also, the necessity for an increased attention in terms of taxon sampling and additional character examinations in certain groups becomes obvious. We herein provide (i) an *R*‐function for automatically translating the character dependency syntax proposed by Grams and Richter (*Cladistics*, 2023, 39, 437) into xlinks‐commands for TNT; and (ii) a TNT‐script for analysing a character matrix successively under various *k*‐values for implied weighting.

## Introduction

Morphological correspondences have been used to systematically group living organisms together even before the advent of evolutionary thinking. Ever since, systematic and later phylogenetic approaches have attempted to find and describe organismal groupings that result in the least contradictory distribution of morphological characteristics, with many of those “ancient” taxonomic categories holding even until today (e.g. Tetrapoda, Mammalia, Aves, Hexapoda). However, early phylogenetic hypotheses, although presented by well‐educated morphologists and taxonomists, and explained in extensive and knowledgeable discussions, were still guided largely by intuition. This changed around the second half of the 20th Century with the growing awareness for cladistic methodology (i.e. phylogenetic systematics sensu Hennig, 1950, 1966) and the increasing amount of morphological knowledge. Morphological characters became more formally conceptualized and their taxonomic distribution summarized in morphological character matrices. Phylogenetic hypotheses explaining the character distributions were henceforth upheld to follow the premise of parsimony with mathematical precision (Farris, 1983). The formulation of character concepts continuously improved, increasing their intelligibility, comparability and adherence to theoretical standards (see Sereno, 2007, for one of the most comprehensive approaches towards a general formalization of character concepts). And while early matrices with comparatively few characters and taxa where still analysed “by hand”, the first cladistic computer programs for the analyses of matrices emerged in the late 1960s (see also Meier, 1992, and citations therein) and ultimately became indispensable for handling the increasing number of characters and taxa included. With the development of molecular methods, morphological approaches seemed to become less and less important, as the analysis of the genetic “code of life” was hoped to be an unbiased and much less time‐consuming alternative. Yet, although genetic analyses are undoubtedly an important accomplishment and a valuable additional tool (especially for morphologically inconspicuous groups or lower taxonomic levels), they are not a proper replacement for morphological phylogenetics. Most obviously, morphological analyses allow the inclusion not only of taxa (yet) unvailable for molecular analyses, such as fossils, but also rare taxa available only as unsuitably preserved museum material and difficult to collect anew (a problem still present for Malacostraca). A second, and probably even more important aspect follows from what at first glance might appear to be a weakness of the morphological approach: The process of character conceptualization (sensu Richter and Wirkner, 2014), a central step towards a morphological analysis, requires an intensive examination and consideration of the analysed groups and characters. Comparable structures have to be identified as (potential) homologues and meaningful degrees of similarity and difference have to be demarcated, not least in the relative context of the taxa included and the previous phylogenetic hypotheses that shall be put to the test. Finally, the computational consequences of specific decisions on character conceptualization (e.g. distinguishing between three instead of four or six states of a character) have to be considered and balanced with all of the other character concepts. Although, of course, time‐consuming, this procedure is highly beneficial for developing a deep understanding of the taxa, their biology and evolution–a step easily omitted for genetic studies, yet fundamental for the final interpretation and causal explanation of the results (see also the preface in Goloboff, 2022). Morphological characters are a representation of the various ideas and hypotheses on homology and evolution, and their cladistic analysis offers not only a phylogenetic tree, but also an evolutionary narrative by outlining the numerous morphological transformations that a specific branching pattern of taxa implies. Of course morphological characters can be and frequently are mapped on molecular trees, yet this procedure strips morphological data of their own phylogenetic value. It instead explains their evolution without actually acknowledging them as an active part in it, ignoring that natural selection first and foremost interacts with the phenotype before indirectly affecting the genotype (cf. Fitzhugh, 2016).

The development of digital matrix management tools such as MorphoBank (O'Leary and Kaufman, 2011; https://morphobank.org), Morph·D·Base (Grobe and Vogt, 2014; www.morphdbase.de) and Mesquite (Maddison and Maddison, 2021) significantly facilitated the creation and (even cooperative) editing of morphological matrices. Moreover, those tools variously allow the inclusion of notes, explanations, literature references and supplementary data (e.g. images, 3D models). This further elevates morphological matrices from being numerically abstracted summaries of observations for the sole purpose of phylogenetic analysis to now multifaceted collections of data, ideas and hypotheses–summarizing and representing the current state of knowledge, while laying the foundation for the next.

## Inapplicables and character dependencies

A common and persistent issue in scoring and especially analysing morphological matrices is the phenomenon of inapplicable characters (“inapplicables”). “Inapplicables” are the result of the encaptic (i.e. hierarchical) structure of organismic morphology and its different levels of granularity (Vogt, 2010; Vogt et al., 2012; Göpel and Richter, 2016). Morphological structures are composed of various (and depending on the perspective and scientific focus variously recognized) substructures on many levels, and comprise numerous properties (e.g. size, colour, shape) that can be the subject of description and comparison. This hierarchy of structures and their substructures and properties leads to ontological dependencies between these describable units. This especially applies to characters and character states, as these represent the comparative summary and conceptualization of those observations. In that context, ontological dependencies become relevant as constraints on the scoring of hierarchically related characters in a morphological matrix and during the optimization process of ancestral state reconstruction — most (in)famously in the form of inapplicability. A character is scored as “inapplicable” when owing to its ontological nature (i.e. its inherent dependency on another character) no actual character state is logically applicable. Maddison (1993) exemplified this phenomenon in his comprehensive discussion on the hypothetical problem of scoring tail colours in the absence of tails in some terminal taxa (i.e. when a character “tail” is scored as “absent” owing to the nonexistence of a tail in a taxon, a character “tail colour” can for this taxon logically only be scored as “inapplicable”). As “inapplicables” were understood and implied to be neutral scorings that do not sway a phylogenetic analysis towards any particular outcome, they appeared similar and even technically identical to the scoring of “missing data” and (at least for binary characters) polymorphisms. Yet, Platnick et al. (1991) already had commented on the conceptual difference of these three scoring options and the importance of distinguishing between them. This was especially proven by Maddison (1993), when he demonstrated on his “tail colour” example that in certain scenarios an “inapplicable” scoring, treated the same as “missing data” (i.e. implying any one of the actual character states to possibly be scored), can lead to questionable outcomes. Specifically, he showed that in cladograms that imply two independent evolutions of a tail, typical parsimony algorithms do favour some phylogenetic hypotheses over others, based only on a reconstruction of tail colour in ancestors reconstructed as not having a tail. Yet, neither the proposed use of “composite coding” (sensu Wilkinson, 1995), nor the various theoretical discussions on “inapplicables” (e.g. Pleijel, 1995; Hawkins et al., 1997; Lee and Bryant, 1999; Strong and Lipscomb, 1999; Seitz et al., 2000; see also the summary in Goloboff, 2022, chapter 3.11) offered sufficient or practical solutions to the problem. De Laet (2005, 2015) was the first to present a viable solution by promoting to follow Farris's (1983) theoretical justification of parsimony, namely the maximization of homology rather than the minimization of transformational steps. To apply the approach of maximizing homology in practice, De Laet (2018, 2020) developed a new algorithm for counting steps of homoplasy in a given reconstruction and implemented it into his cladistic program anagallis. In Goloboff et al. (2021), they extensively discussed the congruence of parsimony with the maximization of homology, developed a more intuitive way of counting nonhomologies, and created an algorithm for the cladistic software TNT that identifies the hierarchical structure of specially labelled characters, automatically rewrites those into composite characters and generates Sankoff matrices for their step costs. And while some limitations still narrowed the practical applicability of these two approaches (see a detailed discussion in Grams and Richter, 2023), both of these software solutions (in anagallis and TNT) represented phenomenal achievements for tackling the issue of inapplicables, and built the foundation for a drastically improved application recently developed and implemented in TNT (with the xlinks‐command, Goloboff and De Laet, 2024; also simply referred to as “xlinks” in the following). Nevertheless, the actual impact and frequency of the problem of “inapplicables” has rarely been tested^1^ has probably been rather underestimated as a niche problem with low occurrence rates in actual datasets. This raises the question of whether this rather theoretical problem justifies the additional work of identifying and analytically including character dependencies and the considerably longer calculation time needed (easily ten‐ to 100‐fold longer) when including “xlinks”. Our phylogenetic analysis of the Malacostraca presented herein also predominantly serves as one of the first applications of this new computational method of handling character dependencies in morphological datasets, testing its utility and significance in cladistics on an actual dataset.

### Conceptualizing serially homologous structures

The phenomenon of (assumed) homology between serial structures within one organism (serial homology sensu Owen, 1848) is highly prevalent all across the Arthropoda (starting with body segments, continuing with the respective appendages of those segments and the various substructures thereof, etc.). Within such a series, singular structures (i.e. morphemes sensu Richter and Wirkner, 2014) can be individualized (“paramorph” sensu Wagner, 2014), enabling their homologization with “the same” structure in other organisms. The identification of corresponding individualized structures in different organisms can be challenging though, especially if the number of structures in a series varies (or if the respective reference system is ambiguous for other reasons, as in the ostia example below). According to Wagner (2014), it is especially difficult in crustacean limbs (as exemplified in an isopod) to judge whether they are sufficiently individualized to represent either paramorphs or rather are unindividualized “homomorphs”. However, in our opinion, at least in Malacostraca the consistent body plan (eight thoracomeres & six pleomeres) enables the recognition of individual identities of each segment and its substructures, so that they generally can be considered as paramorphs. This also allows (for the most part) obvious and convincing homology hypotheses to be inferred and applied for the conceptualization of such serial structures as phylogenetic characters. Nevertheless, the individuality of a serial structure (i.e. a paramorph) does not necessarily mean that it is entirely independent from the evolution and development of other (possibly less individualized) parts in the series. Rather, there can be different degrees of individualization, especially in regards to substructures or certain properties of a serial structure. Although a paramorph can show high individuality in the evolution and development of certain aspects, other aspects may still underlie the same collective identity (and thus evolution and development) of other (homo‐ or paramorph) parts in the series. For those cases of nonindividual and identically repeated transformations in serial structures (independent of being homo‐ or paramorph), we here adopt the term “homomorph” sensu Wagner (2014) and refer to them as “homomorph transformations”.

In the last decades, a central aspect in understanding the evolution and transformation of serial homologues has been the identification of Hox genes and gradual shifts of their expression patterns (e.g. Averof and Patel, 1997; Abzhanov et al., 1999; Abzhanov and Kaufman, 2000a, b; Bruce and Patel, 2020), implying that small genetic changes can affect several serial structures at once. Still, the question of what character conceptualization captures (our idea of) the evolution of these structures most adequately is not easily answered. Creating individual characters for each single structure in the series (and respectively their substructures and properties) might be an intuitive first approach. However, this ignores the partly dependent nature of these structures (a single mutation, or more general “evolutionary step”, may very well affect several or all of these structures at once) and overestimates the evolutionary relevance and “weight” of concurrent homomorph transformations in serial structures. This calculative bias becomes stronger, the more serial structures are encompassed in a series. In Malacostraca, this applies to a certain extent to the series of eight thoracopods and five pleopods and their respective substructures (e.g. endopods, exopods, epipods, oostegites, specific podomeres). For the pleopods, the individual plasticity seems (for the most part) sufficiently low to consider and summarize them (and their substructures) as homogeneous collective characters (e.g. “Pleopods 1–5, endopods: (0) absent, (1) present”). In the series of eight thoracopods, however, much more individual plasticity can be found, most notably in the first pair of thoracopods (Thp1) commonly modified into a so‐called “maxilliped” (but see also the critical re‐discussion of this term and concept in Grams et al., 2023). As a consequence of this high potential of individualization, all characters relating to Thp1 have been considered entirely independent from Thp2–8 in our character matrix. An alternative to the conceptualization of individual characters for every serial homologue is the conceptualization of “pattern characters” summarizing, for example, the distribution pattern of thoracopodal exopods (“Thoracopods 2–8, exopods, distribution pattern: (0) on Thp2–8, […] (3) on Thp2–3, […] (6) on Thp6–8”). However, although this approach does avoid the overestimation of homomorph transformations, it also underestimates or ignores the different grades of similarity between the individual patterns. As a pattern character is a transformational character without an intrinsic order or “distance” of its states, each pattern is treated as equally likely to transform into any other pattern, independent of their specific similarities. This can be solved by using a step matrix for the pattern character, yet such an approach would basically (depending on the specifics of the step matrix) negate the original advantage of the pattern approach. Furthermore, ancestral nodes (and thereby the optimization of character transformation) are limited to being reconstructed as having patterns observed in the terminal taxa (or more precisely to those patterns conceptualized as character states), ignoring unobserved but potentially more parsimonious intermediate patterns. The use of individual characters, however, is automatically gradual and allows the reconstruction of unobserved intermediate patterns.

With the development of the xlinks‐command for character dependencies in TNT, Goloboff and De Laet (2024) also implemented a method of considering the partial dependency of serial homologues, so that the overestimation of co‐occurring homomorph transformations is countered by downweighting their combined step count. This also includes the option of restricting serial transformations to occur in “streaks”, precluding the reconstruction of disrupted patterns of serial homologues. Although this is an additional limiting hypothesis on the evolution of serial homologues imposed on the phylogenetic analysis, it can be a reasonably arguable assumption in many cases. A last problem of using individual characters for serial homologues (even when moderated with “xlinks”) is that the evolutionary emergence (and loss) of such serial structures is reinforced to be gradual from few to many (and vice versa), as nongraduality would imply additional transformational steps. We see no reason to strictly consider the evolutionary appearance of serial homologues to happen like that, especially in the context of substructures of already existing serially homologous structures (e.g. legs or even body segments), where a single transformation/mutation may easily cause modifications (e.g. additional substructures) on several or even all serial homologues. A solution to this problem (other than using pattern characters) is the inclusion of an additional neomorphic character representing the general presence (or absence) of the serial homologues, that acts as a hierarchically higher character (HHC sensu Grams and Richter, 2023) on which the characters for the individual serial structures are dependent on. Consequently, an “absent” (or “inapplicable”) in the HHC causes inapplicability in all the individual characters, whereas a transformation to “present” freely allows the “evolution” of any scoring pattern for the individual characters [at least when analysed under proper dependency consideration, e.g. using “xlinks”; otherwise the original problem described by Maddison (1993) might come into play]. Note that this approach also generally enforces the implication of homology of the serial structures (e.g. oostegites present in entirely different segments in two species are still likely to be resolved as homologous and derived from an ancestral intermediate pattern of oostegites). Whether this ultimately represents a reasonable assumption has of course to be assessed for each individual case. A summary of the implications of the “individual characters” and “pattern” approaches is presented in Table 1. Overall, pattern characters generally seem to create more problems than they solve, so that individual characters are the better choice in most cases, especially when moderated with “xlinks”. In any case, the use of an additional HHC for the general presence/absence of the serial structures is, in our opinion, to be preferred if the serial homology of those structures appears unquestionable.

**Table 1:** Implications for the phylogenetic analysis when conceptualizing serial homologues as either individual absent/present characters or as pattern characters (without additional use of step‐matrices or “xlinks”, unless specifically stated)

Individual absent/present characters	Pattern characters
Always gradual (no step matrix needed)	Only gradual with step matrix, else free pattern transformations
Unobserved intermediate patterns possible in ancestral nodes (also disrupted ones)	Limited to observed (or conceptualized) patterns
Reinforces gradual evolutionary emergence from few to many (unless using an additional general HHC for applicability)	Every (conceptualized) pattern can be the evolutionary starting point
Reinforces gradual evolutionary loss from many over few to none (unless using an additional general HHC for applicability)	Serial structures can be lost in a single mutation/evolutionary step
Less compact (more composite states) for dependency complexes (e.g. with “xlinks”)	More compact (less composite states) for dependency complexes (e.g. with “xlinks”)

Two especially complicated cases of serial homologues are the ostia and cardiac arteries of the heart. Both cases present high diversity within Malacostraca in both number and position, and lack a clearly established positional reference system for their homologization. Hence, neither their number, nor their relative position in either the heart or specific segments of the body can reliably be homologized and satisfactorily conceptualized as characters or states (see also Richter and Scholtz, 2001; Wirkner and Richter, 2010). As an example, imagine the case of a species “A” with a single pair of ostia (OsA) situated most anteriorly in a heart that starts in thoracomere 3 (Th3), and another species “B” with a heart starting in Th1 and two pairs of ostia, one situated in Th1 (OsB1) and one in Th3 (OsB2). Is OsA in species “A” homologous to OsB2 based on its position in Th3 or to OsB1 because it sits anterior‐most in the heart? And how does this relate to three pairs of ostia in Th2, 3 and 4 in a species “C”? As an alternative, conservative pattern character concepts are used in our character matrix that avoid possible mis‐homologizations of ostia or arteries but in turn (owing to the high number of distinguished character states) offer little character informativity beyond the already fairly stable level of taxonomic orders.

## Malacostracan phylogeny

The Malacostraca are a highly diverse (>44 000 species; WoRMS, 2024) and the best‐known taxon of the paraphyletic crustaceans, including well known groups such as Decapoda (e.g. crabs, crayfish, lobsters and decapod shrimps), Euphausiacea (“krill”), Isopoda (e.g. woodlice), Amphipoda (e.g. sandhoppers) and Stomatopoda (mantis shrimps). The monophyly of Malacostraca is strongly supported morphologically by its unique body‐plan: eight‐segmented thorax + six‐segmented pleon (seven only in Leptostraca), with gonopores on thoracomere eight in males and thoracomere six in females. Yet, despite their ecological and economic importance and after more than two centuries of research, their evolutionary history is still riddled with uncertainty in many aspects. Hypotheses on the systematic classification of the malacostracan taxa have been eagerly discussed at least since the end of the 19th Century (e.g. Boas, 1883; Claus, 1886, 1888; Hansen, 1893), with the most controversial debates back then centring around recognizing Leptostraca as malacostracans, and the validity of the Schizopoda as a grouping of Euphausiacea and Mysidacea. Boas (1883) was the first who rejected Schizopoda and introduced Peracarida (yet without naming them). The detailed examinations by Claus (1888) eventually strongly established the Leptostraca as malacostracans, placing them as sister group to Eumalacostraca. Thomson (1894) further extended the Schizopoda by his discovery of *Anaspides tasmaniae*, before Calman (1897, 1899) included it into the hitherto fossil taxon Syncarida and also discussed a close relationship with *Bathynella natans*, discovered shortly before by Vejdovsky (1882). Soon after, Calman (1904) erected a new classification (based on Boas, 1883, and Hansen, 1893) for the Malacostraca that remains widely accepted up to the present day. Most notably, he associated the Euphausiacea with the Decapoda as Eucarida, and grouped the Mysidacea with Isopoda, Amphipoda, Cumacea and Tanaidacea, establishing the Peracarida. In the following decades the Peracarida were further complemented by the discovery of the first representatives of Thermosbaenacea (*Thermosbaena mirabilis* Monod, 1924), Spelaeogriphacea (*Spelaeogriphus lepidops* Gordon, 1957), Mictacea (*Mictocaris halope* Bowman and Iliffe, 1985) and Bochusacea (*Hirsutia bathyalis* Sanders, Hessler & Garner, 1985), although the dorsal brood pouch of Thermosbaenacea (instead of a ventral marsupium, characteristic for Peracarida) has kept its affiliation with the Peracarida controversial to this day.

The first comprehensive hypotheses on malacostracan phylogeny, expressed as phylogenetic trees, started to emerge during the second half of the 20th Century (e.g. Siewing, 1956; Fryer, 1964), supporting in general the classification of Calman (1904, 1909). They showed Stomatopoda as sister taxon to a group containing the monophyla Syncarida, Eucarida and Peracarida (later summarized as Caridoida by Hessler, 1983). Since then malacostracan phylogeny has remained highly debated. Schram (1981), Watling (1983, 1999), Nylund et al. (1987) and Watling et al. (2000) proposed various scenarios for the nonmonophyly of Peracarida, yet successive morphological phylogenies mostly refuted that idea. Wills (1998) presented a phylogenetic analysis of Malacostraca within a broad sampling of extant and fossil crustaceans (yet without Leptostraca), demonstrating monophyly of Malacostraca with paraphyletic Syncarida on its base. His analysis again supported both monophyletic Eucarida and Peracarida, but placed Stomatopoda as sister taxon to the Eucarida. A later analysis (Wills et al., 2009) with focus on Malacostraca showed similar results, but positioned Stomatopoda between Anaspidacea and Bathynellacea, while also showing differently resolved relationships within the Peracarida. A cladistic analysis by Richter and Scholtz (2001), including new anatomical details of the ommatidia, questioned the Eucarida by supporting a closer affinity of Euphausiacea to Syncarida and Peracarida, together forming the Xenommacarida (originally proposed by Richter, 1999). Poore (2005) used a mixture of ground plan and exemplar approach in his analyses by including for the first time also single (fossil and extant) species representing Mictacea and Spelaeogriphacea alongside “established” supraspecific taxa. Major results are the monophyly of Peracarida, including Thermosbaenacea as ingroup peracarids, and the sister‐group relationship of Isopoda and Amphipoda (forming monophyletic Edriophthalma). Wirkner and Richter (2010) provided further support for the phylogenetic hypothesis of Richter and Scholtz (2001) (although without testing Syncarida by leaving out Bathynellacea) by again expanding the character matrix with extensive data on the circulatory system. They also were among the first to apply an exemplar approach (sensu Prendini, 2001) in (morphology‐based) malacostracan phylogeny, preceded only by Wilson (2009) (focusing on isopods and including both, morphological and molecular analyses).

The emergence of genetic analyses facilitated a comparison of malacostracans with other crustaceans, which consistently gave further evidence for the monophyly of Malacostraca, and also for the basal position of Leptostraca (e.g. Giribet et al., 2001; Regier et al., 2005, 2010; Oakley et al., 2013; Noah et al., 2020; Bernot et al., 2023; Yu et al., 2024). Yet, molecular studies constantly disagreed with many of the phylogenetic relationships proposed on the basis of morphology. Most influential was the frequent rejection of monophyletic Mysidacea (e.g. Jarman et al., 2000; Spears et al., 2005; Meland and Willassen, 2007; Jenner et al., 2009; Wilson, 2009; Ashford et al., 2019), mostly by positioning Mysida apart from Lophogastrida and outside Peracarida (rendering Peracarida *de facto* nonmonophyletic as well). Although morphological analyses (e.g. Richter and Scholtz, 2001; Poore, 2005; Wilson, 2009; Wirkner and Richter, 2010; Tabacaru and Danielopol, 2011) constantly contradicted this hypothesis by yielding a monophyletic Mysidacea, the taxon has been widely held as invalidated ever since. The phylogenomic analysis by Schwentner et al. (2018) was the first molecular study to show a monophyletic Peracarida, yet without testing mysidacean monophyly as a consequence of lacking Lophogastrida (and Stygiomysida) in their taxon sampling. Also, it showed neither Eucarida, nor Xenommacarida, but resolved Euphausiacea as sister taxon to Anaspidacea, both together as sister taxon to Decapoda (as also vaguely suggested already by the analyses of Giribet et al., 2005). The mitogenomic analysis by Höpel et al. (2022) was the first molecular study to provide genetic support for the monophyly of Mysidacea (including all three taxa Mysida, Lophogastrida and Stygiomysida), positioned within monophyletic Peracarida. Similar results were shown recently by two phylogenomic analyses of Pancrustacea (Bernot et al., 2023; Yu et al., 2024), yet lacking data for Lophogastrida. As sister taxon to a monophyletic Peracarida Bernot et al. (2023) further proposed the new taxon Stomatocarida, with Stomatopoda as sister taxon to the also new taxon Syneucarida (including both nonmonophyletic Syncarida and Eucarida, with Decapoda as sister taxon to a monophylum of again Anaspidacea and Euphausiacea, and Bathynellacea as basal branch‐off to all three). Yu et al. (2024) generally showed the same results, while also presenting an alternative topology, mostly differing in the position of Stomatopoda again as sister taxon to the remaining Eumalacostraca.

Within Peracarida, the most contentious alternatives relate to the question of whether all taxa with a manca stage form the monophyletic clade Mancoida (“mancoid line” sensu Watling, 1981; Hessler, 1983; see also Richter and Scholtz, 2001; Wirkner and Richter, 2010) or alternatively Isopoda (with a manca stage) and Amphipoda (without manca) are sister groups, forming the Edriophthalma (“those with sessile eyes”), a taxon that goes back to Leach (1815) (see also Poore, 2005). Within Mancoida, traditionally, Tanaidacea has been suggested as sister group to Isopoda (Richter and Scholtz, 2001) but this has been challenged by characters from the circulatory system, which support a sister‐group relationship between Isopoda and Cumacea (Wirkner and Richter, 2010). The latter as well as the Mancoida also has been supported by the phylogenomic analysis of Schwentner et al. (2018). Mancoida are further supported by other recent molecular analyses (e.g. Höpel et al., 2022; Bernot et al., 2023; Yu et al., 2024), although the internal resolution of this taxon is not yet congruent.

Now, although the results of morphological and molecular analyses finally start to converge, many phylogenetic questions remain highly debated. Most cladistic analyses seem to speak against Xenommacarida, yet morphology based analyses are rare and mostly difficult to compare (e.g. having a different taxonomic focus) to that of Wirkner and Richter (2010). Also, the competing hypothesis of Eucarida is not beyond doubt, as the position of Euphausiacea has been resolved differently by various recent analyses. Likewise, the placements of Anaspidacea and Stomatopoda keep changing, as well as the specific relationships within Peracarida. Additionally, lesser‐known but phylogenetically highly interesting taxa such as Bathynellacea, Amphionidacea, Thermosbaenacea, Stygiomysida, Spelaeogriphacea, Mictacea and Bochusacea are still lacking in most molecular analyses, and even “established” groups such as Tanaidacea and Cumacea are still drastically undersampled in otherwise rather comprehensive molecular datasets.

## Objective

Herein we present a comparison of the phylogenetic results produced by traditional methodology (all characters with equal weights, unordered and unpolarized; “inapplicables” as missing data) with those from the more elaborate analytical methods, implied weighting (IW; Goloboff, 1993) and the composite‐character‐step‐matrix‐approach for ontologically dependent characters in TNT (Goloboff et al., 2021; Goloboff and De Laet, 2024). A newly assembled morphological matrix for Malacostraca serves as a practical example for applying these different cladistic methods to a real dataset with a high degree (>67%) of ontological dependencies. This application of different methods (i) represents a kind of sensitivity analysis for the phylogenetic results, evaluating the “stability” of certain alleged monophyla and phylogenetic constellations throughout the different analyses, and (ii) allows a cross‐evaluation of the different (traditional, established and novel) methods themselves.

We herein provide as supplements (i) an *R*‐function for automatically translating the character dependency syntax proposed by Grams and Richter (2023) into xlinks‐commands for TNT v.1.6 (see Goloboff and De Laet, 2024); and (ii) a TNT‐script for analysing a character matrix successively under equal weighting (EW) and various *k*‐values for IW.

## Materials and methods

### Morphological character matrix of Malacostraca

A new morphological matrix for Malacostraca was compiled from the character matrices published by Richter and Scholtz (2001), Poore (2005), Jenner et al. (2009), Wilson (2009), Wirkner and Richter (2010), Tabacaru and Danielopol (2011), and Göpel and Wirkner (2018). Of the synthesized character list, most uninformative characters were excluded and all characters reconceptualized and reformulated according to Sereno (2007), distinguishing two fundamental types of characters (neomorphic and transformational) and consisting of (up to) four components [locator(s), variable, variable qualifier, character states], to ensure “speaking the same character language” (Goloboff and Sereno, 2021). Serial structures (e.g. thoracomeres, pleomeres, thoracopods, pleopods) were numbered, with “1” representing the anterior‐most structure, successively proceeding posterior‐ward. In cases of numbered articles or podomeres of appendages, “1” refers to the most proximal structure, successively proceeding to the most distal. Most characters are qualitative and some quantitative, but in all cases the character states are discrete. The synthesized and new character concepts were aimed at being formulated as morphological as possible, mostly avoiding characterizations based purely on function (e.g. “ventilatory”, “respiratory”, “used for brood care”, “[…] feeding”) and instead referring to the respective morphological structures involved (e.g. “pleopods, oosetae” instead of “brood care with pleopods”). The characters were further conceptualized to strictly follow the Hennigian concept of character states, understanding character states (of the same character) as homologous (conditions of) structures within a transformational series (Hennig, 1966; see also Grant and Kluge, 2004). In total, a list of 207 characters was put together for a taxon sampling of 35 species representative of all 18 currently recognized extant “orders” (sensu WoRMS, 2024). Of these 207 characters, 11 refer to muscles and kinematic aspects, 15 to optical organs, 37 to the circulatory system, eight to the digestive system, 28 to the reproductive system, 15 to developmental data, 87 to general external morphology and another six to other internal structures. The taxon sampling follows the exemplar approach (Mishler, 1994; Yeates, 1995; Prendini, 2001) and was orientated largely on the sampling used in Wirkner and Richter (2010). It was further extended to additionally cover phylogenetically interesting taxa such as Amphionidacea (*Amphionides reynaudii*), Bathynellacea (*Bathynella natans*), Procarididea (*Procaris hawaiana*), Bentheuphausiidae (*Bentheuphausia amblyops*), Stygiomysida (*Spelaeomysis bottazzii*, *Stygiomysis holthuisi*) and Bochusacea (*Thetispelecaris remex*).

The matrix was edited and scored via the online matrix management tool MorphoBank (O'Leary and Kaufman, 2011) (Project 3744; http://morphobank.org/permalink/?P3744), where the character concepts and the specific scoring decisions were documented transparently with literature citations, comments, notes and pictures. This especially includes decisions on scoring taxa based on “proxies” (one or more presumably closely related species or general statements on a superordinate taxon) to substitute otherwise missing information on some characters for that specific terminal taxon (a summarizing list of those “proxy”‐taxa is provided as supplement). Of the 7245 matrix cells, 931 (~12.85%) represent missing data, with *Stygiomysis holthuisi* having the least complete scoring with missing data in 80 characters (~38.65%). Ontological character dependencies (sensu Vogt, 2018; Grams and Richter, 2023), identified among 139 characters (>67%, not counting single state dependencies), were designated at the beginning of the character statements using the syntax proposed in Grams and Richter (2023). Based thereon, respective rules for automated scoring were defined using the “Ontology”‐function in MorphoBank (for practicality reasons mostly limited to negative downstream dependencies; “DSDs” sensu Grams and Richter, 2023). Finally, a tnt‐output file was created using the online infrastructure provided by MorphoBank. For the different analyses two copies of that file (“MatrixNOxlinks.txt” & Matrix_xlinks.txt”; in the following referred to without the file extension “.txt”) were further modified manually [e.g. removal of incompatible quotation marks in character names and inclusion of xlinks‐commands (Goloboff and De Laet, 2024) for dependency consideration in TNT]. For the dataset “Matrix_xlinks” xlinks‐commands were generated by translating the dependency syntax (Grams and Richter, 2023) in the character statements using our “DepSynTrans.R” function (provided in Appendix S1). Additional single‐state dependencies were identified and transcribed manually into another 31 xlinks‐commands in the matrix file (with short explanations added therein). Additionally, three xlinks*‐commands were used to define exopods and oostegites on thoracopods 2–8 (Thp2–8), as well as the form of the endopods on Thp2–5 (no such characters for Thp6–8 are used), respectively, as serially homologous (see “Conceptualizing serially homologous structures” section above). The exopods and oostegites were thereby defined to transform as streaks (excluding the possibility of disrupted distribution patterns). To allow the downweighting of step counts for co‐occurring homomorph transformations in serial homologues with the xlinks*‐command, all characters were upweighted ten‐fold in the dataset “Matrix_xlinks”.

### Ambiguous scoring of pleopodal ramus in *Bathynella natans*

The pleopods in *Bathynella natans* are uniramous, and in some representatives of Bathynellacea even further reduced to setae only. Consequently, the homologous identity of this pleopodal ramus (representing either the endo‐ or exopod) is unclear. Our matrix includes two neomorphic characters for the absence/presence of both the pleopodal endopods and exopods (nos 98 & 102), that moreover are hierarchically higher characters (HHCs sensu Grams and Richter, 2023) to a group of another seven ontologically dependent characters (nos 99–100 & 103–106), which based on the interpretation of the identity of the ramus can be scored as either a specific state or as inapplicable. Thus, an identification of the single ramus as either endo‐ or exopod affects more than just the two character‐scorings. Treating both (or all nine) characters as missing data is ill advised, as this additionally allows for scoring optimizations as either both rami absent or both present, which are definitely false for uniramous pleopods. Regarding the slightly lateral position of the pleopodal ramus and its vague similarity to the thoracopodal exopods, we ultimately decided to score *B. natans* as having pleopodal exopods. The scoring was changed manually after the export from MorphoBank and applied to all analyses.

### Cladistic analyses

The leptostracan *Nebalia bipes* was defined as the outgroup. Cladistic analyses using parsimony as optimality criterion were performed with wTNT 1.6 (Goloboff and Morales, 2023; latest used update: November‐04‐2024). Using our TNT‐script “BrowseKvalues.run” (provided in Appendix S1) separately on the two datasets “MatrixNOxlinks” and “Matrix_xlinks” we performed in total 54 different analyses combining either EW or IW (Goloboff, 1993; using different *k*‐values between 1 and 25) with or without “xlinks” (Goloboff and De Laet, 2024). For the dataset without “xlinks” (“MatrixNOxlinks”) the search command “xmult= rep 20 hits 10 ratchet 100 drift 100 norss noxss nocss; bbreak;” was used. Analyses including “xlinks” (requiring easily 10‐ to 100‐times longer calculation times) were instead performed using “xmult = rep 5 hits 2; bbreak;” to reduce calculation time. The script allows users to perform an equal weighted (“EW”) tree search, followed by a sequential number of “IW” searches defined by the user (in this study we performed IW tree searches using *k*‐values from 1 to 25). Additionally, the script performs an additional IW tree search with an automatically set *k*‐value (in the following “*sk*”) such that a maximum possible ratio *N* for the implied weights cannot exceed a certain value, thus determining the admitted range of weights (Goloboff et al., 2008). By default the script is set to *N* = 15, as also used for the present analyses. This means that a character with zero (0) homoplasy will weigh 15‐fold more than a character with the highest homoplasy found in the dataset. The respective MPTs (“most parsimonious trees”) found for each analysis are saved as separate files. After each of the different analyses, the script additionally includes the found MPTs in a “TreeCollection” (discarding duplicates), and cross‐checks the MPTs found in the current analyses with the trees included in the “TreeCollection” by using a round of TBR (“bbreak;”). Ultimately, this cross‐check loops all analyses (using only TBR with the “TreeCollection”) until no additional trees are found (i.e. added to the “TreeCollection”). This can be seen as an additional check for avoiding local optima, but primarily guarantees the evaluation of the shortest trees found by the other analyses (particularly those with similar *k*‐values). Especially for the “xlinks” analyses this proved to be a time‐efficient approach (<5 min per analysis) for finding additional or even shorter MPTs (thus also allowing to resort to less exhaustive search commands). For each analysis the script generates a strict‐consensus tree from the respective MPTs and calculates the bootstrap support values (we used 1500 pseudo‐replicates for the analyses without “xlinks” and 100 pseudo‐replicates for those with “xlinks”). The “TreeCollection” compiled by the analyses of dataset “MatrixNOxlinks” was used as a starting point for the analyses of the dataset “Matrix_xlinks”. The thereby further extended “TreeCollection” was then used for a re‐run of the analyses of dataset “MatrixNOxlinks” (and vice versa), until no additional MPTs were found (i.e. added to the “TreeCollection”). For those re‐runs the searches were limited to TBR.

In all analyses all characters were treated as unordered and unpolarized (i.e. without predefined or forbidden directions of transformation) and no additional step matrices were used (apart from those for dependency complexes automatically defined in the “background” by “xlinks”).

Tree distances were measured using the normalized Robinson Foulds metric (Robinson and Foulds, 1981), abbreviated in the following as “nRF‐distance” or simply referred to as “distance” (no other distance measures were used). The distances were used as a measure of similarity of the MPTs retrieved from either a single analysis (here referred to as “internal distance”) or from different analyses, and in extension as a measure of congruence among the different analyses. Tree distance heat maps were created using the “TreeDistMatrix.run”‐script from Torres et al. (2022).

## Results

The analysis of dataset “MatrixNOxlinks” under EW resulted in 18 MPTs, that all feature two main branches: a first branch containing Stomatopoda as sister group to monophyletic Eucarida (Decapoda + Euphausiacea, with Amphionidacea as sister group to both); and a second branch with Anaspidacea as sister group to monophyletic Peracarida (however, including Bathynellacea). Within Peracarida, Mysida and Lophogastrida form a monophyletic sister taxon to the remaining Peracarida, with monophyletic Stygiomysida branching off next. Bathynellacea form a monophylum with Thermosbaenacea, together being the sister group to the non‐mysidacean Peracarida, within which first Spelaeogriphacea and then Mictacea branch off next. Bochusacea form the sister group to Tanaidacea, together branching off from the remaining “core” Peracarida, within which Cumacea form either the sister group to monophyletic Edriophthalma (Isopoda + Amphipoda) or to Amphipoda, with then Isopoda being the outgroup to both.

**Fig. 1:**
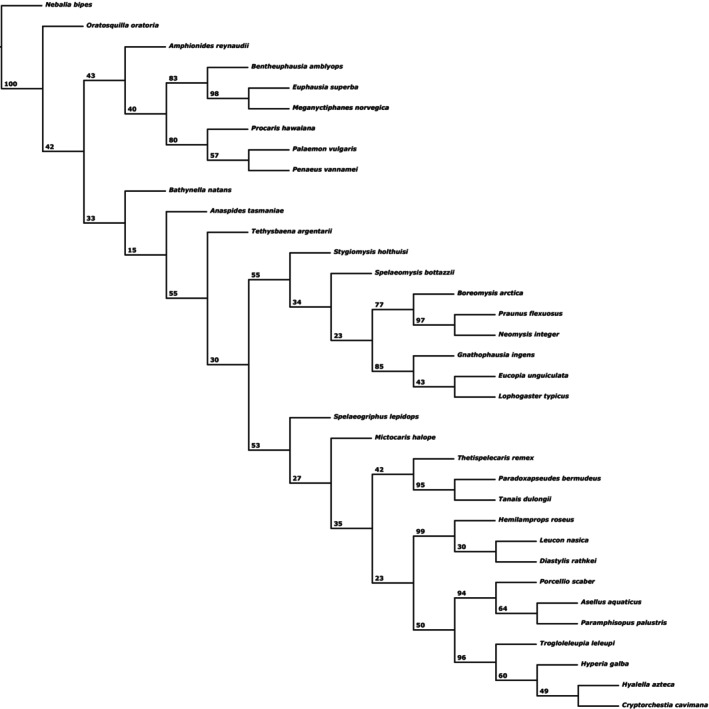
Single MPT from analysing dataset “MatrixNOxlinks” under IW with *sk* = 5.443699. Numbers on nodes indicate bootstrap support.

Analysing the dataset “MatrixNOxlinks” under IW with *sk* (*k* = 5.443699) resulted in a single MPT (Fig. 1). Differently to the EW analysis, Stomatopoda branch off first in the Eumalacostraca, forming the sister taxon to the Caridoida. Bathynellacea is here retrieved as sister group to the monophylum of Anaspidacea + Peracarida. Within Peracarida, Thermosbaenacea form the most basal branch, followed by monophyletic Mysidacea (with paraphyletic Stygiomysida at its base). Isopoda and Amphipoda are resolved as monophyletic Edriophthalma.

Testing additional *k*‐values between 1 and 25 resulted in an additional five different trees. Analyses with *k* ≥ 12 basically confirmed the EW analysis. For *k* ≤ 11, Stomatopoda are the sister group to Caridoida, Mysidacea are monophyletic and Thermosbaenacea are resolved as the most basal branch in Peracarida. Also, Bathynellacea are resolved as either sister group to Anaspidacea (forming monophyletic Syncarida) or to Anaspidacea + Peracarida. Only for *k* = 1 do Bathynellacea form the sister group to all Eumalacostraca. Also, the Eucarida are no longer resolved as monophyletic and instead the Euphausiacea are placed as sister group to Anaspidacea, together forming the sister group to Peracarida, thus forming monophyletic Xenommacarida. Furthermore, Amphipoda are then resolved as sister group to now monophyletic Mancoida, including monophyletic Cosinzeneacea Guțu (1998) (Spelaeogriphacea + Mictacea) as sister group to Isopoda. The phylogenetic results of EW and different *k*‐values for IW are summarized in Table 2.

**Table 2:** Summary of the different results from analysing dataset “MatrixNOxlinks” under EW and IW with *k*‐values ranging from 25 to 1

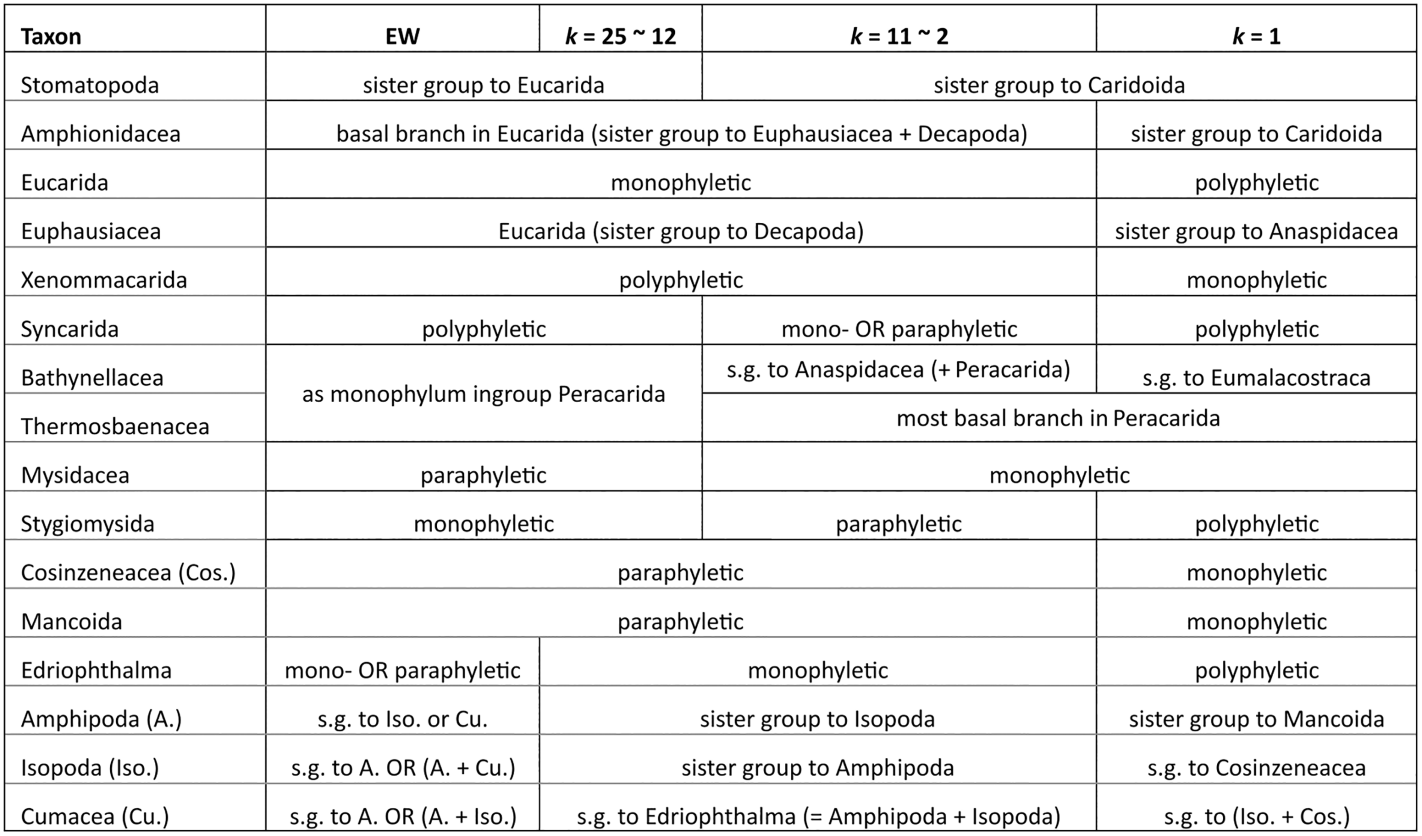

a., Amphipoda; Cos., Cosinzeneacea; Cu., Cumacea; e., Edriophthalma; I., Isopoda; s.g., sister group.

**Fig. 2:**
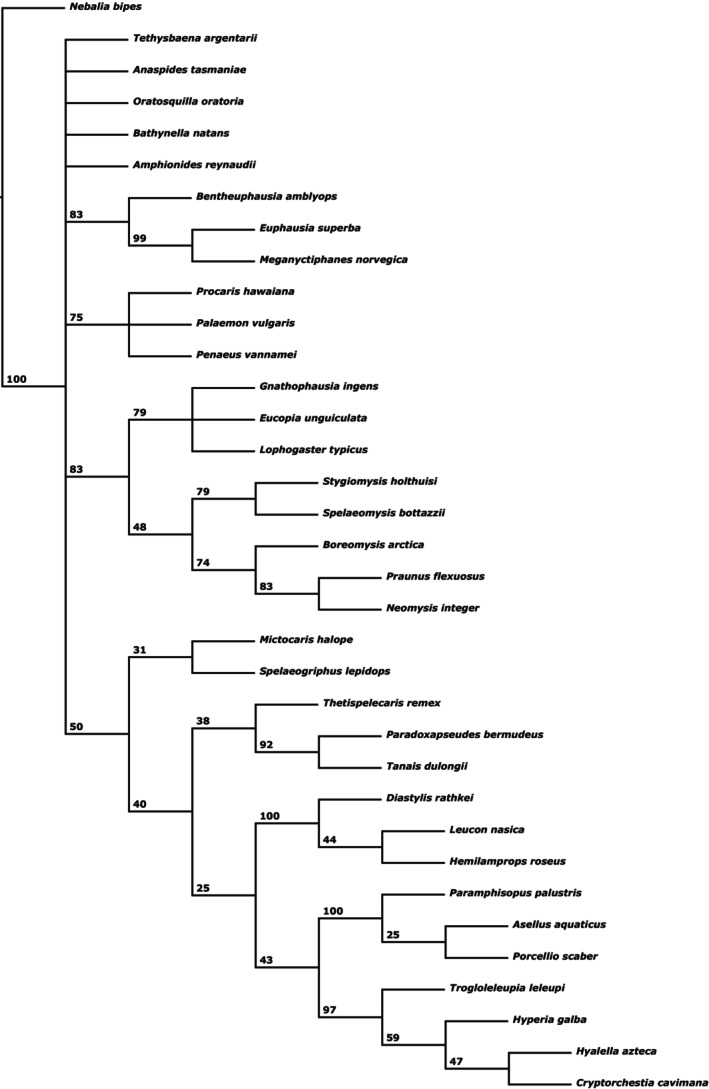
Strict consensus trees of the eight MPTs obtained from the “xlinks” analysis under EW. Numbers on nodes indicate bootstrap support.

**Fig. 3:**
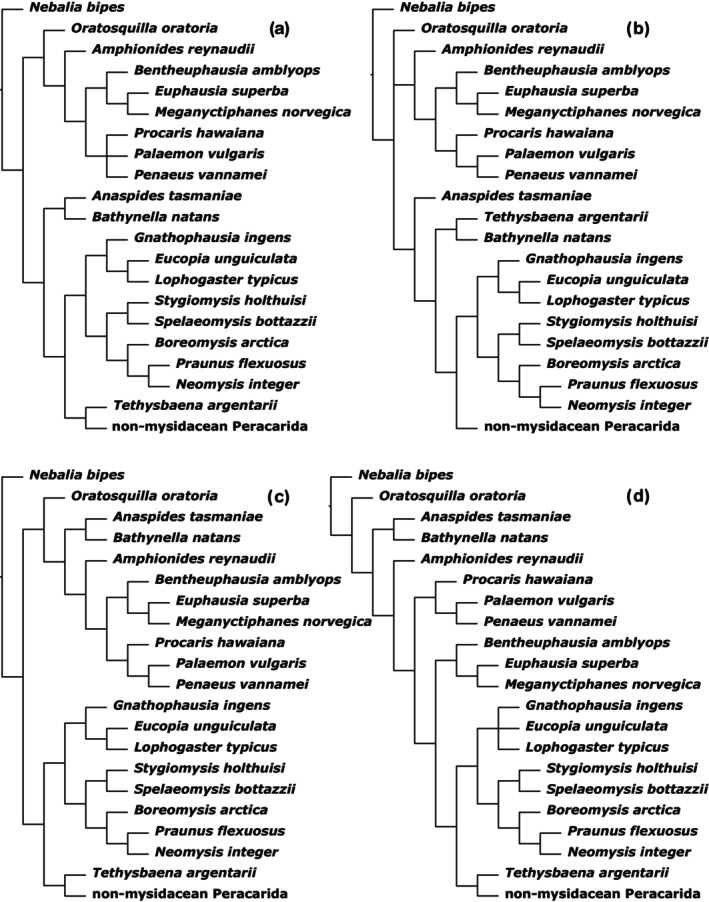
Strict consensus trees (CTrees) of subgroups of the eight MPTs obtained from the “xlinks” analysis under EW, showing the four main topologies found: (a) CTree of two MPTs (nos 1–2); (b) CTree of three other MPTs (nos 3–5); (c) Single MPT (no. 6); and (d) CTree of two other MPTs (nos 7–8). Non‐mysidacean Peracarida are summarized here as they were resolved identically (same topology, see Fig. 2) in all eight MPTs.

The analysis of dataset “Matrix_xlinks” under EW resulted in eight MPTs with four major topologies (strict consensus tree shown in Fig. 2). All eight MPTs support the monophyly of Peracarida, with monophyletic Mysidacea as basal branch and monophyletic Cosinzeneacea as sister group to the “core” Peracarida, within which Cumacea form the sister group to monophyletic Edriophthalma. Differences concern mostly the positions of Stomatopoda, Anaspidacea, Bathynellacea and Thermosbaenacea, and also the monophyly of Eucarida. Stomatopoda is resolved either as sister group to Eucarida (Fig. 3a,b), Eucarida + Syncarida (Fig. 3c), Caridoida (Fig. 3b,d) or the non‐eucaridan Eumalacostraca (Fig. 3b). Eucarida are mostly resolved as monophyletic (Fig. 3a–c), but also as a paraphyletic grade in two MPTs (Fig. 3d). Five MPTs resolve Syncarida as monophyletic, being positioned as sister group to either Eucarida (Fig. 3c), Peracarida (Fig. 3a) or to the remaining Caridoida (Fig. 3d). The other three MPTs (Fig. 3b) separate Anaspidacea from Bathynellacea and instead resolve the latter as the sister group to Thermosbaenacea, together forming the sister group to Peracarida. In the five trees with monophyletic Syncarida (Fig. 3a,c,d), Thermosbaenacea are placed within Peracarida, as sister group to the non‐mysidacean peracarids.

Analysing the dataset “Matrix_xlinks” under IW with *k*‐values between 1 and 25 resulted in an additional three different MPTs (see also the summary in Table 3). Analyses with *k* ≥ 10 recovered one of the eight MPTs also found by the “xlinks” EW analysis (one of the two MPTs represented in Fig. 3d). A different MPT was found for *k* = 4–9 (incl. *sk* = 5.443699), with a similar topology to Fig. 3b, but resolving Syncarida as monophyletic and Stomatopoda as sister group to Syncarida + Peracarida. IW with *k* = 2–3 found one MPT similar to the one shown in Fig. 3c, but with Amphionidacea not being part of Eucarida and with Bathynellacea separated from Anaspidacea and instead forming a monophylum with Thermosbaenacea, as sister group to Peracarida. Additionally, for the entire range of *k* = 2–9, the internal resolution of Peracarida includes a change that now resolves Cumacea as sister group to [Tanaidacea+Bochusacea]. Most changes are involved in the MPT retrieved under IW with *k* = 1. Its topology somewhat resembles the result of *k* = 4–9, but with Amphionidacea not resolved as part of Eucarida and with Bathynellacea placed as the sister group to all Eumalacostraca. Also, the internal resolution of Peracarida now features Mancoida instead of Edriophthalma.

**Table 3:** Summary of the different results from analysing dataset “Matrix_xlinks” under IW with *k*‐values ranging from 25 to 1

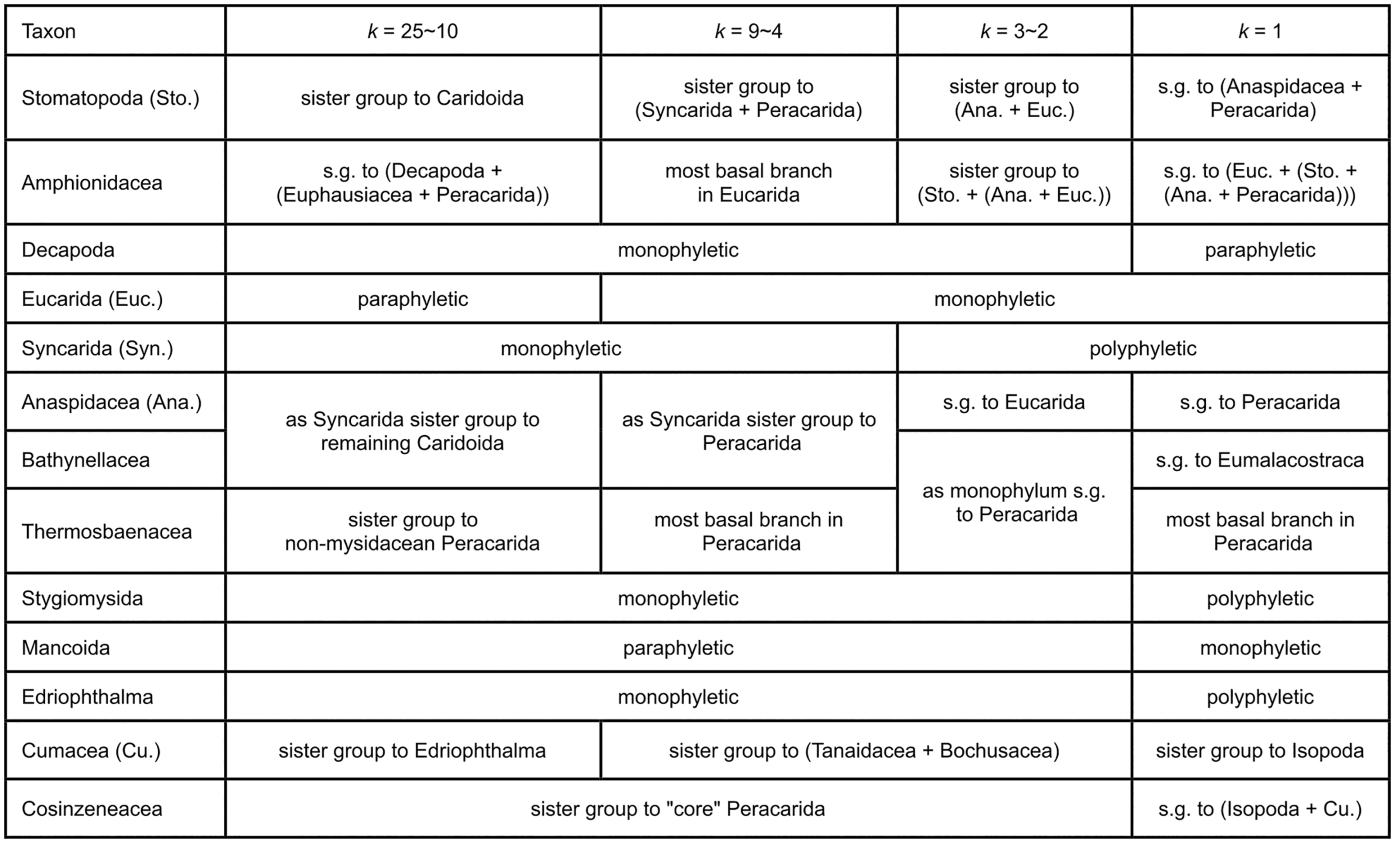

Ana., Anaspidacea; Cu., Cumacea; D., Decapoda; Euc., Eucarida; s.g., sister group; Sto., Stomatopoda; Syn., Syncarida.

Repeating the analyses with a modified version of dataset “Matrix_xlinks” that does not treat the serial homologous characters (thoracopodal exopods and oostegites) as “streaks” (i.e. only transforming as coherent distribution patterns, avoiding disrupted patterns) resulted in the exact same MPTs, however with considerably longer calculation time.

## Total comparison using nRF‐distance

**Fig. 4:**
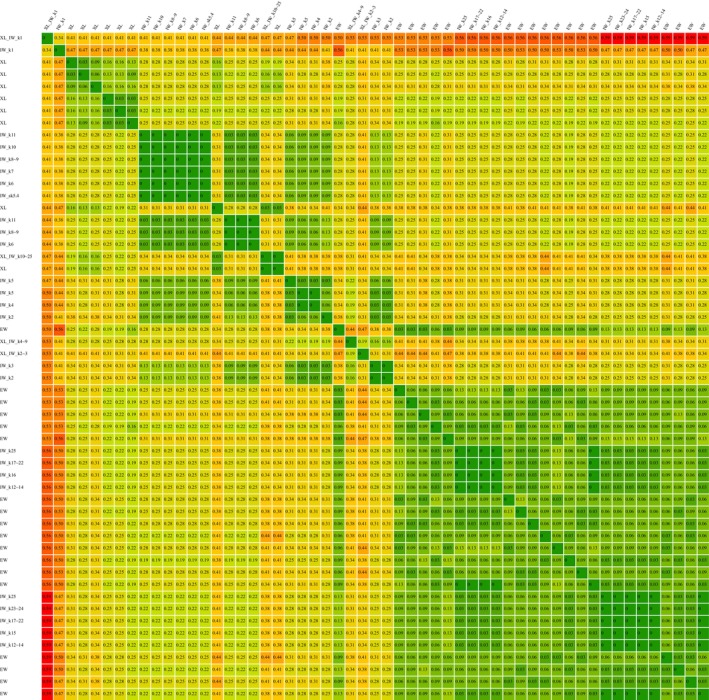
nRF‐distances between all MPTs from all 54 analyses. The distance matrix is “rooted” on the most extreme and singular tree, obtained from the xlinks analyses under IW *k* = 1, meaning the order of all other MPTs is based on their respective nRF‐distance to that singular MPT. The distance matrix is available as svg‐file in the supplement. EW, equal weighting; IW, implied weighting; XL, “xlinks” analysis.

**Fig. 5:**
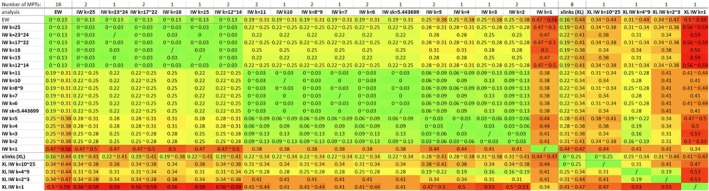
Summary of the nRF‐distances of all obtained MPTs clustered by analyses. The table is available as xlsx‐file in the supplement. EW, equal weighting; IW, implied weighting; XL, “xlinks” analysis.

In total the 54 analyses resulted in 36 different (respective) MPTs. In an overall comparison of these 36 different MPTs (Fig. 4, summarized in Fig. 5), the largest nRF‐distance of 0.59 is found between the single MPT from the “xlinks” analysis under IW *k* = 1, and four MPTs found without “xlinks” under EW or IW with *k* = 12–25. In general, the two MPTs for IW *k* = 1 (with and without “xlinks”) are either the most distant or among the most distant to the MPTs from all other analyses (with the sole exception of both being closest to each other, yet at a moderate distance of 0.34).

Of the 25 MPTs retrieved from the 27 analyses without “xlinks” (in the following called “traditional”), none were identical to the 11 MPTs found when using “xlinks”. The “internal” nRF‐distance for (i.e. between the 18 MPTs resulting from) the traditional EW analysis is fairly low (max. 0.13). Unsurprisingly, the nRF‐distances of those 18 MPTs to those of the respective IW analyses are greater the lower the *k*‐value used. Although the analyses with *k* ≥ 12 still yielded (exclusively) two MPTs also found under EW, analyses with *k* ≤ 11 resulted in seven new trees. In comparison, the “xlinks” EW analysis shows a moderate internal distance of maximally 0.25. Using “xlinks” with IW *k* ≥ 10 yielded a single MPT also found under EW, whereas analyses with *k* ≤ 9 resulted in three new trees. Again expectedly, the nRF‐distances of the eight “xlinks” EW MPTs to those of the respective IW analyses are greater the lower the *k*‐value used. When comparing the eight MPTs from the EW “xlinks” analysis with the 18 MPTs from the traditional EW analysis, the nRF‐distance ranges from somewhat similar (0.16) to rather different (0.44). Interestingly, the three “xlinks” EW MPTs (those summarized in the consensus tree in Fig. 3b) closest (0.16–0.19) to nine of the traditional EW MPTs also include the one “xlinks” EW MPT closest (0.22) to the traditional *sk*‐IW analysis. In general, the MPTs from the “xlinks” EW analysis show a moderate distance (0.22–0.34) to the MPT(s) from the traditional IW analyses under *sk* = 5.443699 – *k* = 11. Compared to the light IW analyses (*k* = 12–25) the distances cover a broader range of similarity (0.19–0.41), whereas compared to the stricter IW analyses (*k* = 2–5) the distances are getting slightly larger (0.28–0.41).

When combining “xlinks” with IW the results deviate even more from those of a traditional EW analysis (with the closest distances being 0.31–0.44 between traditional EW and “xlinks” IW with *k* = 4–9). Distances of “xlinks” IW MPTs (for *k* = 2–25) are slightly smaller (0.31–0.38) to the two MPTs from the traditional analyses under light IW (*k* = 12–25). Tree distances are getting even smaller, especially for the “xlinks” MPT from moderate IW (*k* = 4–9, including *sk*), when compared to the traditional analysis under moderate IW (*sk* = 5.443699 ~ *k* = 11, distances 0.25–0.28) and especially stronger IW (*k* = 2–5, distances 0.16–0.19). The closest two trees in this comparison with a distance of 0.16 are the “xlinks” MPT from IW with *k* = 4–9 and the MPT from traditional IW with *k* = 3.

## Discussion

Compiling morphological matrices for such a diverse and disparate taxon as Malacostraca faces the problem of what could be called the “morphological impediment”. Whereas molecular tools can be applied for various taxa on various hierarchical levels, compiling morphological matrices always requires at least some kind of taxon‐specific knowledge. This refers to the terminology as well as the study of the morphemes (sensu Richter and Wirkner, 2014; Göpel and Richter, 2016). Experts on many taxa or organ systems, however, are scarce (and increasingly so). And while the external morphology is for the most part comparatively easily accessible, either by direct study using, for example, various kinds of light microscopy or alternatively by using taxonomic literature, the inclusion of internal anatomical or even developmental characters is already more challenging, needing either more sophisticated technology (e.g. micro‐computed tomography, confocal laser scanning micrsocopy, transmission electron microscopy, immunohistochemistry) or studies with living animals. In any case, the importance of a thorough character conceptualization (Rieppel and Kearney, 2002; Richter, 2005) has to be emphasized, although sometimes ignored by the assumption that the number of characters included might be more important than the quality of each character conceptualization.

On a philosophical level, we do not and will not know the “true phylogeny” (a simple fact often ignored), so the question remains which of the analyses (if any) is to be preferred and based on what criteria? We do not follow the nowadays commonly held notion that phylogenetic hypotheses based on molecular data are always to be preferred to morphological ones, with some researchers even going so far as to suggest that morphology has nothing to contribute to phylogenetics at all (e.g. Sharma et al., 2021; Ballesteros et al., 2022; Gainett et al., 2024).

Nevertheless, congruence between analyses using morphological data with those using molecular data could certainly be a measure of confidence in certain phylogenetic relationships. This can be seen as an *extrinsic* approach to judge the reliability of a certain analytical method (e.g. as used in our study). However, this is only the case if the datasets are indeed independent from each other, as for the comparison of phylogenomic approaches with morphological ones. In the case of successive morphological analyses, often using (in part) the same characters, congruence with analyses from previous studies cannot be considered as support for a certain analytical approach, as similar datasets can unsurprisingly lead to similar results. Therefore, we do not compare our results with those from previous morphological analyses (but see Introduction > Malacostracan phylogeny).

So, the question raised is whether any *intrinsic* attributes of the different analytic approaches make them superior to others. Equal weighting of all characters and analysing them based on Fitch parsimony approaches the problem of not knowing the individual quality (in terms of complexity and/or susceptibility to convergence) of each character nor the direction of evolution by arguing against *a priori* weighting and against Dollo parsimony and step matrices, thereby enforcing the premise that all characters are equally reliable (see also the discussion in Goloboff, 2014). However, this premise is far from reality and even careful character conceptualization does not exclude the presence of convergences. For that reason, the introduction of implied weighing by Goloboff (1993) was a big step forward in analysing morphological characters, and in our view IW is indeed superior to EW, as also has been demonstrated on simulations by Goloboff et al. (2018) and recently on genealogies of actual datasets by Ezcurra (2024). However, the consequent and more complicated question is which *k*‐value is to be preferred. While our “BrowseKvalues.run” script makes it easy to examine a broad range of *k*‐values, thus avoiding having to choose one or few specific *k*‐values, it is nevertheless useful to have some sort of orientation for narrowing down a relevant spectrum of *k*‐values for a more detailed evaluation. Automatically defining an “*sk*”‐value by TNT (based on the conclusions from Goloboff et al., 2008) can certainly serve this purpose. Also, as pointed out already in previous discussions of IW (e.g. Goloboff et al., 2018), the use of too strong IW (i.e. too low *k*‐values) is not advisable.

Another arguably *intrinsically* superior method (compared to a simple EW analysis) is the consideration of ontological character dependencies, for example through the use of xlinks‐commands in TNT (Goloboff and De Laet, 2024). As discussed above (and known since Maddison, 1993), the treatment of inapplicable characters (owing to character dependencies) as “missing data” is problematic. However, the actual impact and frequency of this problem had so far remained almost untested and the use of “xlinks” (or comparable methods) requires additional theoretical and practical preparation of the dataset and considerably longer calculation time (easily ten‐ to 100‐fold), raising the question of whether this additional work is actually worthwhile. Our results now show considerable nRF‐distances (≤0.44; see Figs 4, 5) between the MPTs from EW analyses with and without “xlinks”, thus demonstrating that character dependencies can indeed have a significant impact on the outcome of a phylogenetic analysis. Moreover, by accepting analyses under IW (especially with *sk* or similar *k*‐values) as an objective improvement over EW analyses, we can use the results of those analyses as a reference for a comparative evaluation of the application of “xlinks”. For our dataset, the eight EW MPTs found with “xlinks” are in the majority of tree comparisons (>75%) either closer (>54%) or at least equally close (>21%) to the single MPT from (traditional) *sk*‐IW than to any of the 18 traditional EW MPTs (whereas in <24% of tree comparisons “xlinks” EW MPTs were closer to traditional EW MPTs than to the traditional *sk*‐IW MPT). Although some “xlinks” EW MPTs are as close as 0.16 to traditional EW MPTs and thus closer to traditional EW than to traditional *sk*‐IW (minimal distance of 0.22), the average distance of “xlinks” EW MPTs to the traditional EW MPTs is slightly greater (~0.29) than the average distance to the traditional *sk*‐IW MPT (~0.27). Thus, we can show that the use of “xlinks” not only generates MPTs considerably different from those of a simple EW analysis, but also shows the tendency of being closer to the results of IW analyses. Of course, all of these observations are based only on the analyses of our single character matrix (and some precursors thereof) and can thus not necessarily be generalized without a broader study of additional independent datasets. Still, given the entirely ontological nature of hierarchical character dependencies (see also Grams and Richter, 2023), the use of dependency considerations (e.g. with “xlinks”) follows a highly logical rationale. Consequently and especially together with the implications of our results, dependency considerations can arguably be seen as a new prerequisite for performing a sound analysis.^2^

**Fig. 6:**
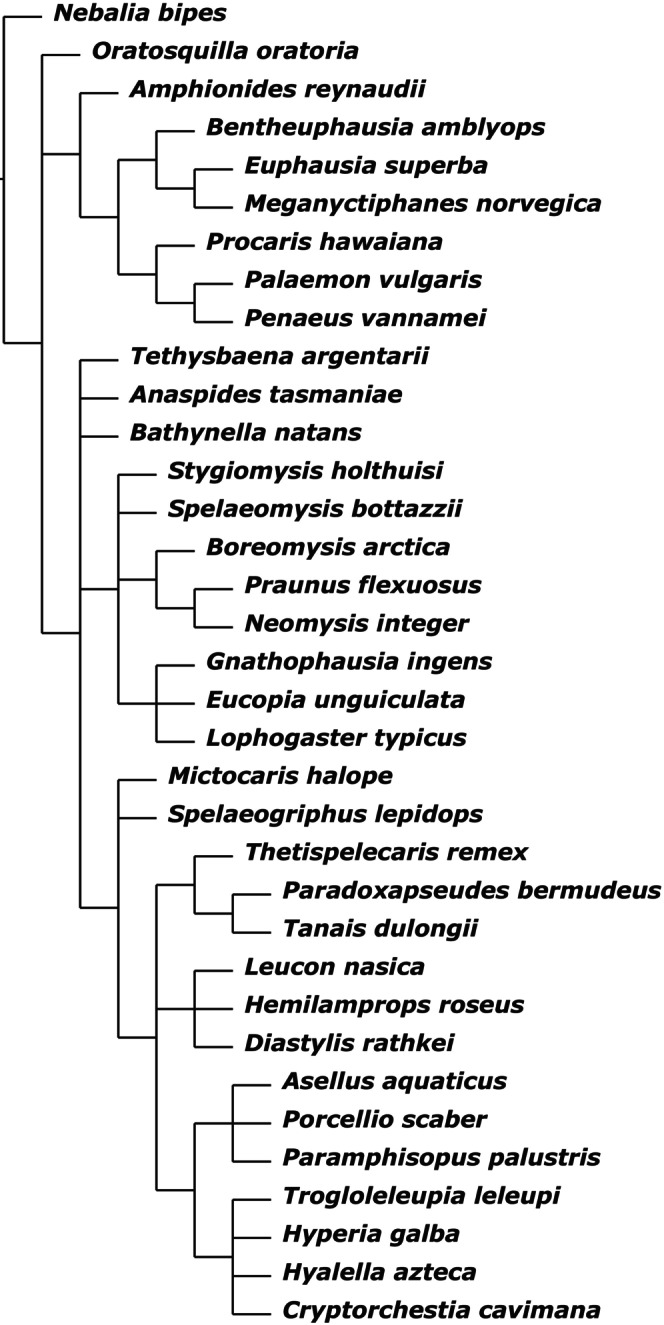
Strict consensus tree of the five MPTs representing the closest nRF‐distances between the two *sk*‐IW analyses (with and without “xlinks”) and the “xlinks” EW analysis.

Of course then, the combination of “xlinks” and IW is obviously a promising option. However, although possible, there are some caveats to be aware of (as already pointed out by Goloboff et al., 2021, and Goloboff and De Laet, 2024). Essentially, the combination of complexes of dependent characters into composite characters complicates the determination of the homoplasy scores in the single characters. Instead, every composite character (representing a character dependency complex) receives a singular homoplasy score based on the averaged homoplasy of its constituent characters, multiplied by their number. Nevertheless, homoplasy of nondependent characters will still be down‐weighted accurately (i.e. as without “xlinks”). And even if the averaged homoplasy score of composite characters only represents an approximation, one might argue that an approximation is better than nothing. So, all three approaches have their strengths and weaknesses: IW alone lacks dependency consideration, “xlinks” alone lacks homoplasy downweighting, and their combination only uses averaged approximations of homoplasy scores for dependency complexes. Hence, arguably none is objectively better than the others and we might consider all of their MPTs as valid results. One way of combining the strengths of all three approaches without necessarily having to include all of their MPTs is to select those MPTs that have the most congruence (i.e. the shortest distance) among the three approaches and combine them into a strict consensus tree (Fig. 6 combines the two MPTs from the *sk*‐IW analyses with and without “xlinks” with those three “xlinks” EW MPTs closest to either *sk*‐IW analysis). Although this obviously almost guarantees unresolved areas of the cladogram, it at least offers an objective way of choosing from the potentially rather diverse pool of alternative MPTs.

## Summary of the phylogenetic results and comparison with those of recent molecular analyses

Throughout our analyses we found fairly consistent support for the monophyly of Eucarida, Peracarida, Mysidacea and the “core Peracarida”‐group (Amphipoda, Isopoda, Tanaidacea, Cumacea; including Bochusacea as sister group to Tanaidacea). More unstable were the positions of Stomatopoda, Anaspidacea, Bathynellacea and Thermosbaenacea, and the internal resolution within the “core Peracarida”. Considering the latter, most of our analyses resulted in a monophyletic Edriophthalma (Isopoda + Amphipoda), a taxon based mostly on reductive similarities (e.g. missing carapace, sessile eyes, missing thoracopodal exopods) and nowadays mostly considered as outdated. Its general rebuttal also is largely founded in its rare retrieval by the phylogenetic analyses (especially not by molecular ones) of the last three decades. The much more widely accepted hypothesis of a monophyletic Mancoida (see the recent genetic analyses by Schwentner et al., 2018; Höpel et al., 2022; Bernot et al., 2023; Yu et al., 2024) was only retrieved in our analyses (both with and without “xlinks”) under very strict IW with *k* = 1. However, despite the consistent retrieval of Edriophthalma in our analyses, the qualitative support for this taxon is not high. Although our matrix contains about 17 characters with mostly congruent scoring between Isopoda and Amphipoda, most do not serve as convincing synapomorphies for this taxon, either representing reductions and “(pseudo‐)homologous absences” or plesiomorphies. Then again, synapomorphies (especially “strong” ones) for alternative relationships within the “core Peracarida” are scarce, demonstrating the need for further studies on comparative morphology (and especially internal anatomy) of these taxa. Xenommacarida was only retrieved with the dataset “MatrixNOxlinks” when analysed under very strict IW with *k* = 1. Regarding the different positions retrieved for both Anaspidacea and Stomatopoda, at least two of the 11 MPTs from our “xlinks” analyses (one from EW and a second from IW with *k* = 2–3) include clades similar to Syneucarida (Bathynellacea + Anaspidacea + Euphausiacea + Decapoda) and Stomatocarida (Stomatopoda + Syneucarida) newly proposed by the recent phylogenomic study of Bernot et al. (2023) and also supported in Yu et al. (2024). Much more frequently, however, our analyses support a close relation of Anaspidacea (or even Syncarida) to Peracarida. The recent tentative interpretation (not considered in our analyses) of coxal endites in female *Anaspides* spp. as potential “proto‐oostegites” (Grams and Richter, 2021) fits in well with this result. The retrieval of Bathynellacea deeply nested within Peracarida, as found in the analyses without “xlinks” under either light IW (*k* ≥ 12) or EW, can probably be ascribed to “mis‐homologizations” of many of the reduced features (e.g. simplified thoracopods and circulatory system, missing eyes, reduced number of pleopods) of Bathynellacea with those of the cavernicolous and still rather understudied peracaridan taxa Stygiomysida, Spelaeogriphacea and Mictacea (partly also Bochusacea). The fairly persistent “phylogenetic adherence” of Bathynellacea and Thermosbaenacea in our analyses also follows this pattern, but at least when resolved as basal branch(es) to Peracarida, their position seems somewhat more plausible. Lastly, the consistent retrieval of Mysidacea in all analyses including either “xlinks” or IW with *k* ≤ 11 (or both) again demonstrates the consistent morphological support for this currently widely rejected group. In this regard our results also agree with the most recent molecular studies (Höpel et al., 2022; Bernot et al., 2023), although even comprehensive molecular studies such as Schwentner et al. (2018), Bernot et al. (2023) and Yu et al. (2024) were still lacking a sufficient sampling of mysidacean species (ideally comprising representatives of Mysida, Lophogastrida, Stygiomysidae and Lepidomysidae) to properly be able to answer the question of mysidacean monophyly from a molecular viewpoint.

Remarkably, some phylogenetic constellations such as the monophyly of Mancoida, the sister‐group relationships of Isopoda & Cumacea, and of Euphausiacea & Anaspidacea, that were retrieved in our analyses only under very strict IW with *k* = 1 (partly with, partly without “xlinks”), have not only all been part of the results of the most recent phylogenomic analyses (Schwentner et al., 2018; Bernot et al., 2023; Yu et al., 2024), but also correspond with the topology suggested in Wirkner and Richter (2010) (however, their with Euphausiacea + Anaspidacea being a paraphyletic grade). This might show that those relationships are supported in our analyses by a few stable apomorphies but these are strongly concealed by several homoplasies.

In any case, the phylogeny of Malacostraca certainly cannot be regarded as solved by our analyses, as is evident already from the several differences in the topologies we found. Much more important and interesting, however, are the methodological implications of our results, which underline again that a morphological analysis has much more complexity to be considered beyond just the topology resulting from it. We are confident that in the future further light will be thrown on the relationship between morphology and phylogenomics.

## Conflict of interest

None declared.

## Supporting information


**Appendix S1.** The presented TNT script and R‐function, both with instructional and example files, as well as other supplementary material (e.g., matrix files, tree files, distance matrices).

## Data Availability

All data of this study are openly available in “Zenodo” at https://doi.org/10.5281/zenodo.15115498. The character matrix is openly available in “Morphobank” at http://morphobank.org/permalink/?P3744.

## Footnotes

1 A recent exception being Smith and Dhungana (2022) with their reanalysis of a fossil Ecdysozoa dataset, by applying the ‘inapplicable‐aware’ algorithm by Brazeau et al. (2019); see also the critical discussion of that algorithm in Goloboff et al. (2021).

2 Similar conclusions have been reached just recently by Urrea et al. (2025) through a very similar analysis of 45 rhizome characters (~50% with character dependencies) of a dataset of 14 extant and 118 fossil fern taxa.
