# Supplementary material for: A new morphological phylogeny of Malacostraca comparing the application of character dependencies and implied weighting
Source: Cladistics. 2025 Apr 4;41(3):283–303. doi: 10.1111/cla.12611 (PMC12065123; doi:10.1111/cla.12611)
Supplement: Supplementary file 1 — Appendix S1. The presented TNT script and R‐function, both with instructional and example files, as well as other supplementary material (e.g., matrix files, tree files, distance matrices). [file CLA-41-283-s001.zip › supplement/scripts/DepSynTrans/DepSynTrans _ Step-by-Step instruction.docx]

**How to use the “DepSynTrans.R” script for automatic translation of the Grams & Richter (2023) syntax for character dependencies into “xlinks” commands (Goloboff & De Laet, 2024) for TNT 1.6 (Goloboff & Morales, 2023):**

Prepare your characters

- Include the character dependency syntax proposed by Grams & Richter (2023) into the beginning of the character statements of HLCs (hierarchically lower characters) of your character matrix
- begin the character statements with the number of the respective character (included before the dependency syntax)
- copy your character statements into a separate “.txt”-file (e.g. “characters.txt”, included as an input example in the Supplement of Grams et al., 2025)

Use the script

- in “RStudio” press [ALT]+[S] and/or go to the upper tool-bar to “Session” > “Set Working Directory” > “Choose Directory...” and select the folder that contains your “characters.txt” file
  - alternatively Press [CTRL]+[SHIFT ]+[H] ...” and select the folder that contains your “characters.txt” file
- make sure the selected folder also contains the **DepSynTrans.R** script; if not, copy it into the folder
- type into the console of “RStudio”:

source("DepSynTrans.R")

- press enter
- type into the console of “RStudio”:

DepSynTrans("characters.txt", "Output.txt")

[“characters.txt” here works as a placeholder for your input-file; you have to include the name of **YOUR** input-file; it has to be a “.txt”-file though]

[if you wish to have a different name for your output-file, you can write that name instead of “Output.txt”; it has to remain a “.txt”-file though]

- press enter
- you will get additional error-notifications if your dependency syntax contains severe mistakes
- in case of a successful run of the script, the “Output.txt” file will have been saved in the selected folder (the same that contains the “characters.txt” file and the DepSynTrans.R script)
- copy the lines from the Output.txt file into your TNT-file that contains your character matrix and/or the commands for the phylogenetic analysis

References:

Goloboff, P.A., De Laet, J. 2024. Farewell to the requirement for character independence: phylogenetic methods to incorporate different types of dependence between characters. Cladistics. 40, 209–241. doi:10.1111/cla.12564

Goloboff, P.A., Morales, M.E. 2023. TNT version 1.6, with a graphical interface for MacOS and Linux, including new routines in parallel. Cladistics. 1–10. doi:10.1111/cla.12524

Grams, M., Richter, S. 2023. On the four complementary aspects of hierarchical character relationships and their bearing on scoring constraints, expressed in a new syntax for character dependencies. Cladistics. 39, 437–455. doi:10.1111/cla.12550
